# Ten years of antibiotic consumption in ambulatory care: Trends in prescribing practice and antibiotic resistance in Austria

**DOI:** 10.1186/1471-2334-9-61

**Published:** 2009-05-13

**Authors:** Sigrid Metz-Gercek, Andreas Maieron, Reinhild Strauß, Peter Wieninger, Petra Apfalter, Helmut Mittermayer

**Affiliations:** 1 Dept. of Hygiene, Microbiology and Tropical Medicine, Elisabethinen Hospital Linz, Fadingerstraße 1, 4010 Linz, Austria; 2 Internal Medicine IV, Dept. of Gastroenterology and Hepatology, Elisabethinen Hospital Linz, Fadingerstraße 1, 4010 Linz, Austria; 3 Federal Ministry of Health, Family and Youth, Radetzkystraße 2, 1030 Wien, Austria; 4 Main Association of Austrian Social Security Institutions, Kundmanngasse 21, 1030 Wien, Austria

## Abstract

**Background:**

The primary aims of this study were (i) to determine the quantity and pattern of antibiotic use in Austria between 1998 and 2007 and (ii) to analyze antibiotic resistance rates in relation to antibiotic consumption in important clinical situations in order to provide data for empirical therapeutic regimens for key indications.

**Methods:**

Consumption data and resistance data were obtained via the Austrian surveillance networks European Antimicrobial Resistance Surveillance System (EARSS) and European Surveillance on Antimicrobial Consumption (ESAC). The EARSS collects data on isolates from blood and cerebrospinal fluid obtained predominantly in the hospital setting. The Anatomical Therapeutic Chemical (ATC) classification and the Defined Daily Dose (DDD) measurement units were assigned to the data. The number of DDDs and packages per 1,000 inhabitants (PID) were used to calculate the level of antibiotic consumption. Antibiotic resistance was expressed in resistance rates, i.e., the percentage of resistant isolates compared to all isolates of one bacterial species.

**Results:**

The overall antibiotic consumption measured in DIDs increased by 10% between 1998 and 2007, whereas PIDs decreased by 3%. The consumption of substances within the drug utilization 90% segment (measured in PID) increased for ciprofloxacin (+118.9), clindamycin (+76.3), amoxicillin/clavulanic acid (+61.9%), cefpodoxime (+31.6), azithromycin (+24.7); and decreased for erythromycin (-79.5%), trimethoprim (-56,1%), norfloxacin (-48.8%), doxycycline (-44.6), cefaclor (-35.1%), penicillin (-34.0%), amoxicillin (-22.5), minocycline (-21.9%) and clarithromycin (-9.9%). Starting in 2001, an increase in the percentage of invasive E. coli isolates resistant to aminopenicillins (from 35% to 53%), fluoroquinolones (from 7% to 25.5%) and third-generation cephalosporins (from 0% to 8.8%) was observed. The percentage of nonsusceptible or intermediate penicillin-resistant pneumococcal isolates remained stable over this time period at around 5%. For macrolides, the rate of resistant isolates increased from 5% to 12.8%, with a peak in 2005 at 14.7%.

**Conclusion:**

The Austrian resistance data can not explain the fundamental change in prescribing practice. The more frequent use of ciprofloxacin has most likely contributed to rising resistance rates in E. coli in Austria. Penicillin G is still a highly effective substance for the treatment of invasive infections caused by pneumococci.

## Background

Antibiotic resistance has become an important public health issue in the last decade. To better understand national, regional, and local trends, it is important to critically assess national data on antibiotic consumption. Moreover, given the increasing rates of antibiotic resistance, it is important to be aware of the relationship between antibiotic consumption and emergence of resistance. Since only a very small number of new antibiotics are under development, physicians can not rely on new drugs alone to treat infections caused by multidrug-resistant bacteria, but must also introduce policies to reduce the emergence and spread of resistant bacteria [1].

With the launch of the European projects EARSS (European Antimicrobial Resistance Surveillance System, 1999) and ESAC (European Surveillance on Antimicrobial Consumption, 2001), comprehensive surveillance of antibiotic resistance and consumption on a national level began in Austria 10 years ago. Since then, the quality and quantity of data has steadily improved. Now it is possible to draw conclusions and shed some light on the actual situation.

The primary aims of this study were (i) to determine the quantity and pattern of antibiotic use in Austria between 1998 and 2007 and (ii) to analyze antibiotic resistance rates in relation to antibiotic consumption in important clinical situations in order to provide data for empirical therapeutic regimens for key indications.

## Methods

### Consumption data

In Austria, antibiotics are available only with a prescription issued by a physician and are dispensed by pharmacies. Antibiotics account for only about 3.7% of the total drug budget and therefore play a minor role in the surveillance of drug prescribing in ambulatory care. For physicians in ambulatory care, the few existing restrictions in antibiotic prescribing are limited mainly to expensive antibiotics such as gentamycin, tobramycin, telithromycin and linezolid.

Quarterly data from the ambulatory care (AC) sector were obtained from the Main Association of Austrian Social Security Institutions (HV) for the purpose of data collection carried out by the European Surveillance of Antimicrobial Consumption (ESAC). The HV is responsible for collecting information on all drugs dispensed and reimbursed by the social health insurance institutions. In Austria, there is no private market for antibiotics and only a very small amount, estimated to be 2%, of antibiotics are cheaper than the reimbursement fee and are therefore not included in the national data. Only a few small packages of tetracycline and trimethoprim are cheaper than the reimbursement fee.

### Units of measurement and approach of analysis

The Anatomical Therapeutic Chemical (ATC) classification and the Defined Daily Dose (DDD) measurement units (ATC/DDD version 2007) were assigned to the data. Besides analyzing the consumption data in DDDs per 1,000 inhabitants per day (DID), the number of packages per 1,000 inhabitants per day (PID) was used as a proxy for prescriptions to measure time trends on a national level, thereby allowing international comparison. Despite the merits of the ATC/DDD classification system, there is a need for a simple and unbiased unit of measurement that disregards changes in package size or changes in dosing [2]. Therefore, prescriptions per 1,000 inhabitants per day were calculated and analyzed in the present study. After assigning the ATC/DDD classification, the 90% drug utilization (DU90; i.e., substances constituting 90% of consumption), expressed as PID and DID, was calculated for all substances to identify those most frequently used. These substances were then further analysed comparing DID and PID as well as evaluating consumption trends over time.

### Resistance data

The Austrian EARSS has the most comprehensive collection of data on antibiotic resistance in Austria and consists of a voluntary network of 41 microbiology laboratories covering more than 85% of all Austrian acute care hospital beds. The number of laboratories participating in this study increased steadily from 10 in 2000 to 41 in 2007. For this study, Pneumococci and E. coli strains causing invasive infections were selected as key organisms for measuring antibiotic resistance. E. coli is the leading cause of community-onset bacteraemia, whereby 68% of infections are community-acquired, 19% are hospital-acquired, and 13% are healthcare-associated (e.g., catheterised patients), but it can still be used as an indicator for antibiotic resistance levels and trends in outpatient care, even if it is measured via hospital data [3]. This also holds true for invasive pneumococcal disease. Data on antibiotic resistance of E. coli against aminopenicillins, fluoroquinolones and third-generation cephalosporines, as well as data on resistance of pneumococci against penicillin and macrolides are routinely collected in accordance with the EARSS protocol [4]. Once a year, an external quality assurance exercise organized by EARSS und provided by the United Kingdom National External Quality Assessment Service (UKNEQAS) is performed in all laboratories reporting to EARSS in Austria [5].

Antibiotic resistance was expressed in resistance rates, i.e., the percentage of resistant isolates compared to all isolates of one bacterial species. The designation of nonsusceptibility was based on the Clinical and Laboratory Standards Institute breakpoints according to the most recent document available at the time. Since breakpoints for the antibiotics analyzed in this study have not changed over the relevant time period, resistance data remain current [6]. According to a consensus decision in 1998, all Austrian microbiology laboratories use CLSI (Clinical Laboratory Standards Institute, Wayne, Pa., USA, formerly NCCLS, National Committee on Clinical Laboratory Standards) standards for interpreting susceptibility test results. The methods used are the disk diffusion test according to CLSI (Performance Standards for Antimicrobial Disk Susceptibility Tests, Document M02 in the respective edition), the E-test (AB Biodisk, Solna, Sweden), both on Mueller-Hinton agar, and the automated systems Vitek 2 (bioMérieux, Marcy l'Etoile, France), Phoenix (BD, Franklin Lakes, NJ, USA) and MicroScan WalkAway (Siemens Healthcare Diagnostics, Eschborn, Germany).

## Results

### Antibiotic consumption

The overall antibiotic consumption measured in DIDs showed an increase of 10% between 1998 and 2007, whereas PIDs decreased by 3%. The number of DIDs ranged from 12.3 – 14.6 and the number of PIDs ranged from 1.7 – 2.0 over time. In the latest available data for 2007, prescriptions for betalactams, macrolides, quinolones, tetracyclines and others accounted for 48.2%, 30.9%, 12.3%, 5.1%, and 3.5%, respectively. In the DID ranking, the most frequently used substance class was betalactams (7.92 DID) followed by macrolides (3.5 DID), quinolones (1.43 DID), tetracyclines (1.27 DID), sulfonamides/trimethoprim (0.31 DID), and others (0.185 DID).

The core group of substances was determined using the DU90 values for 1998 and 2007. An overview of the substances included in the DU90 segment of 1998 and 2007 as well as PID and DID values and changes of PID and DID in percent of 1998 are displayed in Table 1. The consumption of substances within the DU90 segment, measured in PID, increased for ciprofloxacin (+118.9), clindamycin (+76.3), amoxicillin/clavulanic acid (+61.9%), cefpodoxime (+31.6), and azithromycin (+24.7); and decreased for erythromycin (-79.5%), trimethoprim (-56.1%), norfloxacin (-48.8%), doxycycline (-44.6), cefaclor (-35.1%), penicillin (-34.0%), amoxicillin (-22.5), minocycline (-21.9%), and clarithromycin (-9.9%). In 1998, trimethoprim, erythromycin, ofloxacin and norfloxacin were included in the DU90 list; moxifloxacin was not included in the DU90 list because it was introduced on the market in 2001. In 2007, its consumption accounted for 2.4% of the total amount used (expressed in PID).

**Table 1:** DID and PID values of 1998 and 2007 as well as change in percent of 1998

Year of DU90	ATC code/Substance name	1998 PID	2007 PID	ΔPID	1998 DID	2007 DID	ΔDID
1998 & 2007	J01AA02 doxycycline	0.11	0.06	-44.63	1.43	1.01	-29.20
1998 & 2007	J01AA08 minocycline	0.04	0.03	-21.87	0.33	0.26	-20.84
1998 & 2007	J01CA04 amoxicillin	0.12	0.09	-22.51	1.15	1.45	26.36
1998 & 2007	J01CE02 phenoxymethylpenicillin	0.19	0.12	-34.01	1.49	1.01	-32.59
1998 & 2007	J01CR02 amoxicillin und enzyme inhibitor	0.24	0.39	61.86	1.65	3.74	126.74
1998 & 2007	J01DB01 cefalexin	0.06	0.05	-7.41	0.29	0.28	-2.81
1998 & 2007	J01DC02 cefuroxime	0.02	0.03	98.15	0.20	0.41	106.56
1998 & 2007	J01DC04 cefaclor	0.09	0.06	-35.13	0.34	0.20	-40.27
1998 & 2007	J01DD08 cefixime	0.09	0.08	-10.06	0.47	0.43	-9.99
1998 & 2007	J01DD13 cefpodoxime	0.05	0.07	31.63	0.23	0.32	36.24
1998	J01EA01 trimethoprim	0.05	0.02	-56.08	0.32	0.20	-37.70
1998	J01FA01 erythromycin	0.04	0.01	-79.45	0.21	0.05	-75.60
1998 & 2007	J01FA09 clarithromycin	0.28	0.26	-9.90	1.89	2.04	7.96
1998 & 2007	J01FA10 azithromycin	0.10	0.13	24.70	0.44	0.48	7.46
1998 & 2007	J01FF01 clindamycin	0.07	0.13	76.73	0.28	0.57	106.95
1998	J01MA01 ofloxacin	0.03	0.01	-62.18	0.22	0.08	-63.45
1998 & 2007	J01MA02 ciprofloxacin	0.06	0.13	118.88	0.35	0.74	110.11
2007	J01MA14 moxifloxacin	0.00	0.05	-	0.00	0.30	-
1998	J01MA06 norfloxacin	0.06	0.03	-48.83	0.43	0.20	-45.78

Δ change in PID or DID in % of 1998

When examining consumption over time, it is noteworthy that there is a considerable divergence between DID and PID values for clarithromycin, amoxicillin, amoxicillin/clavulanic acid, clindamycin, azithromycin, and doxycycline. To explain this discrepancy, the average grams of substance per package were determined. The analysis showed that indeed, the average package size of these substances has changed over the study period. For example, the average grams per package of amoxicillin/clavulanic acid increased from 5.1 to 9 grams during this time.

### Antibiotic resistance

**Figure 1:**
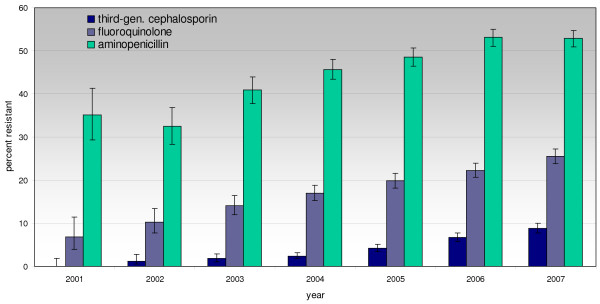
**Percentage of resistance to fluoroquinolones, third-generation cephalosporines and aminopenicillins in E. coli isolated from blood (2001: n = 260; 2007: n = 2,606)**.

**Figure 2:**
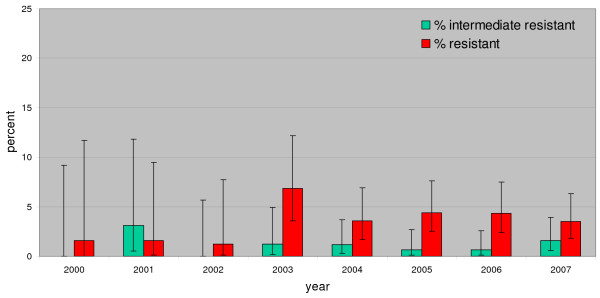
**Percentage of high-level and low-level resistance to penicillin in invasive Pneumococci in Austria 2000–2007 (2000: n = 63; 2007: N = 323)**.

**Figure 3:**
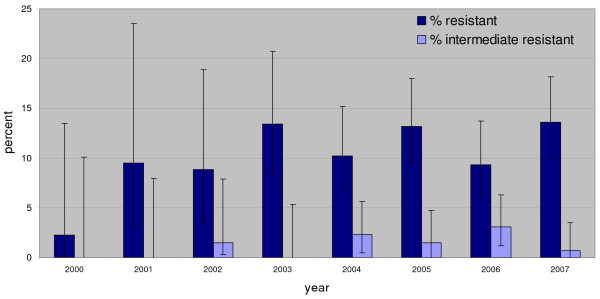
**Percentage of high-level and low-level resistance to macrolides in invasive Pneumococci in Austria 2000–2007 (2000: n = 63; 2007: N = 323)**.

Since 2001, a continuous and steep increase in the percentage of invasive E. coli isolates resistant to aminopenicillins (from 35% to 53%), fluoroquinolones (from 7% to 25.5%) and third-generation cephalosporins (from 0% to 8.8%) was observed. In 2001, 61.1% of all isolates were susceptible to all four substance classes, but this number decreased to 41.9% in 2007. In addition, the rate of isolates resistant to more than one substance class increased from 8.1% in 2001 to 25.5% in 2007. The percentage of invasive E. coli resistant to all three substances increased from 1% to 8% over time. The percentage of nonsusceptible or intermediate penicillin resistant pneumococcal isolates remained stable over time at around 5%. In 2007, five isolates showed high-level resistance to penicillin. For macrolides, the rate of resistant isolates increased from 5% to 12.8%, with a peak in 2005 at 14.7%. The resistance rates for invasive E. coli and Pneumococci are shown in Figures 1, 2 and 3.

## Discussion

Analyzing antibiotic consumption data is a difficult task. Although it is agreed that unified classification systems and measurements such as DDDs are needed for international comparison, there are still unresolved issues concerning how to measure change over time within one country accurately and how to retrieve the values closest to reality. By looking only at consumption expressed in DDD, change in the consumption patterns could be over- as well as underestimated if the average grams per package change over time. The use of packages as a proxy for prescriptions seems to be appropriate to avoid this bias.

Despite all technical difficulties, surveillance of antimicrobial use is a key strategy to monitor appropriateness of antimicrobial therapy. In Austria, consumption is relatively low (12.3 – 14.6 DID) in comparison to other European countries (mean DID 19.04) and the USA (24.9 DID) [7]. In Europe, the level of consumption ranges from 9.8 DID in The Netherlands to 33.4 in Greece. In the past ten years, the level of antibiotic consumption has not changed substantially, regardless of the measurement used. Compared to other European countries, Austria ranges among the low consumer countries such as The Netherlands, Germany and Estonia for penicillins as well as for total consumption [8,9]. With regard to cephalosporins and fluoroquinolones, Austria ranges mid-field in Europe, meaning a relatively high proportion of fluoroquinolones and cephalosporin compared to other European countries [10,11]. Although the total volume of consumption has not changed over time, the composition of substances did. There was a considerable increase in the consumption of fluoroquinolones, betalactams and a decrease in the use of tetracyclines and sulfonamide/trimethoprim. The level of consumption of macrolides did not change over time.

Analysis of the consumption of particular substances shows that especially the use of ciprofloxacin and amoxicillin/clavulanic acid has increased dramatically, indicating that substances with a lower selective pressure, such as penicillin, have been exchanged for amoxicillin/clavulanic acid [8]. The percentage of use of moxifloxacin has increased continuously, from 15% in 2004 to 20% of total fluoroquinolone use in 2007 [11]. In 2001, the patent for ciprofloxacin expired and since then, the number of prescriptions has more than doubled. Interestingly, the increase of ciprofloxacin prescriptions is almost equal to the magnitude of the decrease in prescriptions of norfloxacin and sulfonamide/trimethoprim together, indicating a switch in the therapy of urinary tract infections in primary care. The increased use of fluoroquinolones and the traditionally frequent use of third-generation cephalosporines [10,12] are very likely to cause and promote the incidence of resistant microorganisms in Austria [13]. It is remarkable that the resistance rate of invasive E. coli to fluoroquinolones rose from 7% in 2001 to 25.5% in 2007; resistance to third-generation cephalosporins also rose from 0% in 2000 to 7% in 2007. Ena et al. showed that the use of fluoroquinolones and urinary catheterisation are independent risk factors for the acquisition of urinary tract infections with ESBL-producing E. coli [14]. This and other evidence in the literature suggest a close association between the use of fluoroquinolones and the increase in the incidence of multidrug-resistant E. coli [15,16].

Routinely-collected surveillance data on antibiotic resistance usually do not include those from uncomplicated urinary tract infections. Due to the lack of representative surveillance data from urinary samples, data on invasive infections must be used for the development of empirical therapy regimens, although resistance might be overestimated, since 32% of E. coli bacteraemia cases are health-care associated or even hospital-acquired. With regard to complicated urinary tract infections, there is evidence from the US that first-line therapy with sulfonamide/trimethoprim might be inadequate due to resistance rates of up to 24%; therefore, it is recommended to use ciprofloxacin instead [17]. In Austria, the rate of sulfonamide/trimethoprim resistance is known only from local data. In our hospital for example, the resistance rate in urinary samples was 23.3% for sulfonamide/trimethoprim and 15% for fluoroquinolones in 2006. Fluoroquinolone resistance in invasive E. coli isolates is high, with up to one third of the isolates being resistant; therefore, fluoroquinolones can not be recommended for first-line therapy. Furthermore, a study from Italy shows that resistance to fluoroquinolones in E. coli has a negative impact on the outcome of community-acquired urinary tract infections (UTI) and their use in this indication should therefore be avoided [18].

S. pneumoniae remains the most important pathogen for community-acquired pneumoniae [19]. The susceptibility results of invasive pneumococci indicate that penicillin G is still very effective, since there is no upwards trend and the number of isolates with high-level resistance is very low (5/323). On the other hand, the macrolide resistance rates do not show a very favourable situation: 15% of all invasive pneumococci isolates were resistant to macrolides. Compared to Germany, where up to 28% of prescribed substances are narrow-spectrum penicillins [8], in Austria, this substance group accounts for only 7% of prescriptions. According to Garcia-Suarez et al., resistance rates in invasive S. pneumoniae are lower than resistance rates in non-invasive isolates, which is why these data should be interpreted with care [20].

### Limitations of the study

Although the increase in fluoroquinolone consumption and the increase of fluoroquinolone resistance coincide, which suggests an association, the results should be interpreted with care. For one, this is an ecological study that considers only aggregated country data for consumption and resistance. Another reason is that it is not possible to make a distinction between hospital and community-acquired infections, which might lead to overestimation of resistance rates. Using only resistance data from hospitals and not taking into account resistance data from the community might add to any overestimation of resistance rates. Furthermore, the lack of consumption data from the hospital setting neglects the possible influence of hospital prescribing on the evolution of resistance.

## Conclusion

In Austria, the level of antibiotic consumption in ambulatory care has remained stable over the last decade. The pattern of consumption, however, has changed dramatically, with a switch from narrow- to broad-spectrum drugs, e.g., replacement of sulfonamide/trimethoprim with ciprofloxacin and penicillin or amoxicillin with amoxicillin/clavulanic acid. The Austrian resistance data can not explain the fundamental change in prescribing practice. The increased use of ciprofloxacin has most likely contributed to rising resistance rates in E. coli in Austria. Penicillin G is still a highly effective substance for the treatment of invasive infections caused by pneumococci.

## Competing interests

The authors declare that they have no competing interests.

## Authors' contributions

SM, AM and HM designed the study. SM, AM, RS, PW, PA, HM collected and assembled data, SM and AM carried out the data analysis and the data interpretation. SM and AM wrote the manuscript. The manuscript was finally approved by all authors.

## Pre-publication history

The pre-publication history for this paper can be accessed here:

http://www.biomedcentral.com/1471-2334/9/61/prepub
